# Epidemiologic and clinical features of multisystem atrophy: a population-based study in Navarre, Spain

**DOI:** 10.1007/s00415-024-12561-4

**Published:** 2024-08-13

**Authors:** M. E. Erro, P. Arrondo, I. Gastón, P. Clavero, J. Sánchez Ruiz de Gordoa, G. Martí Andrés, R. Valentí, J. Delfrade, E. Vicente

**Affiliations:** 1 Department of Neurology, Hospital Universitario de Navarra, C/Irunlarrea 3, 31008 Pamplona, Navarra Spain; 2 Epigenetic Group, NavarraBiomed, Pamplona, Spain; 3 Navarra Institute for Health Research (IdisNA), Pamplona, Spain; 4 Department of Neurology, Clínica San Miguel, Pamplona, Spain; 5 Navarra Community Health Observatory Section, (ISPLN), Pamplona, Spain; 6 CIBER Epidemiology and Public Health (CIBERESP), Madrid, Spain

**Keywords:** Multisystem atrophy, Atypical Parkinsonism, Epidemiology, Incidence, Prevalence, Survival, Time to diagnosis

## Abstract

**Background:**

Epidemiological studies on multisystem atrophy (MSA) are scarce. Our aim has been to analyse 10-year incidence, point prevalence, survival, and the time to diagnosis of MSA in Navarre, a northern Spanish region.

**Methods:**

This is a population-based observational retrospective study, from 2012 to 2021, which covered the population of Navarre (followed until 31 December 2021). Data from various sources of health information were reviewed in order to identify all potential diagnoses of MSA, that were validated from medical records. Patients were included if they fulfilled the new Movement Disorder Society criteria.

**Results:**

We observed a crude average annual incidence rate (IR) of 0.49/100,000 person-years, with the highest occurring in the age group of 60–69 years. No significant IR differences by sex or subtype were observed. Point prevalence in December 2021 was 2.43/100,000 inhabitants. Joinpoint analysis for global incidence and prevalence experienced stable annual rates during the whole period, showing an upward trend for prevalence without a statistically significant slop. The median age at symptom onset was 65 years (range 47–76). The median time to diagnosis was 36 months, without statistically significant differences between sex, age at diagnosis or subtypes. Median time of survival from clinical onset was 7 years. Age of onset above 70 years and autonomic onset were associated with reduced survival.

**Conclusions:**

This is the first population-based epidemiological study on MSA in Spain. It provides detailed incidence and prevalence data for MSA that may be useful for appropriate management of health resources.

**Supplementary Information:**

The online version contains supplementary material available at 10.1007/s00415-024-12561-4.

## Introduction

Multisystem atrophy is a rare sporadic and progressive neurodegenerative disease that manifests clinically with a variable combination of parkinsonism, cerebellar dysfunction, and/or autonomic failure [1]. Two motor subtypes are distinguished: MSA-parkinsonian type (MSA-P) and MSA-cerebellar type (MSA-C), according to predominant motor features [2]. The neuropathological basis consists of striatonigral and pontocerebellar degeneration accompanied by oligodendroglial α-synuclein deposits [3]. MSA diagnosis is based on clinical criteria (Online Resource 1) which have been recently updated [4].

There are not many epidemiological studies on MSA, mostly conducted in Europe (Table 1) and US, and there is lack of studies specifically examining the epidemiology of MSA in Spain [5–7]. Recent review studies have reported an incidence of MSA of 1 cases/100,000 person-years and a prevalence around 10 cases/100,000 persons [7, 8]. The age of onset has been generally estimated between late 50 s and the early 60 s, without significant gender differences. In general, survival time of MSA from disease onset is lower than 10 years [8].

**Table 1:** Epidemiological population-based studies on multisystem atrophy

	Geographic area	Population	Period (years)	Number of cases (M/F)	Crude prevalence (per 10^5^ persons)/adjusted incidence (per person-years)
Linder [32]	Umea˚, Sweden	141,950	4 (2004–2007)	12 (8/4)	I 2.4
Winter [33]	Moscow, Russia	1,237,900	2.5 (2006–2008)	4 (1/3)	I 0.11
Osaki [34]	Kochi, Japan	66,465	1 (2007)	11 (9/2)	P 17
Savica [21]	Olmsted County, USA	141,360	15 (1991–2005)	15 (11/4)	I 0,8
Bjornsdottir [18]	Iceland	319,368	10 (1999–2009)	16 (10/6)	P 3.1 I 0.7
Caslake [22]	Aberdeen, Scotland, UK	317,357	3 (2002–2009)	17 (14/3)	I (MSA-P) 1.4
Fleury [5]	Canton of Geneva, Switzerland	470,512	10 (2009–2012)	19 (8/11)	P 4.3 I 0.6
Kaplan [6]	USA	331,893,745	6 (2016–2021)	1502 (714/788)	P 7.2 I 14.2
Present study	Navarre (Spain)	664,117	10 (2012–2021)	40 (24/16)	P 2,43 I 0,55

*F* female, *I* incidence, *M* male, *MSA-P* multisystem atrophy Parkinsonian subtype, *P* prevalence

The aim of our study has been to perform an epidemiological study of MSA in Navarre (a northern Spanish region) for a 10-year period (2012–2021) and to analyse incidence, point prevalence, survival, and time to diagnosis in our region.

## Material and methods

### Study population

This is a population-based observational retrospective study, from January 2012 to December 2021, which covered the population of Navarre, one of the 17 Autonomous Communities (AC) in northern Spain. The population of Navarre in 2022 was 664,117 inhabitants (49.5% women), comprising the 1.4% of the Spanish population [9].

The Spanish National Health System (S-NHS) is based on the principles of universality, free access, equity, and fairness of financing, and is mainly funded by taxes [10]. Over 98% of Navarre’s citizens have an individual health card with a unique 8-digit personal identification code (called CIPNA), which allows them to have access to the public health system. It contains information on birth date, sex, and other socio-demographic conditions, and enables linking across databases through a unique identifier.

Neurologist ratio per inhabitant in Navarre is 4.65/100,000 inhabitants [11]. Since 2010, movement disorders consultations attended by specialized neurologists have been implemented in the Navarre University Hospital, the single large tertiary referral hospital for the entire region, which receives referrals from the entire community. A multidisciplinary care strategy has been in place for the management of atypical Parkinsonism since 2019.

### Data sources

Navarre has hosted a population-based Rare Disease Registry (RERNA) since 2013 [12], which uses all available Health Information Systems (HIS), in addition to medical reporting, to record both retrospectively and prospectively, prevalent cases from 2000. The HIS used by RERNA to collect MSA cases were: Minimum Basic Data Set at Hospital Discharge, Temporary Work Disability Registry, and Mortality Statistics [13–15]. To get the maximum sensitivity in identifying MSA cases, all International Classification diseases-10 diagnostic codes used for any type of MSA were included: G23.2 (striatonigral degeneration), G23.8 (other specified degenerative diseases of basal ganglia), G23.9 (degenerative disease of basal ganglia, unspecified) and G90.3 (multisystem degeneration). In addition, the Electronic Clinical Records from all Neurology hospital Departments (including inpatients, outpatients, and all private hospitals) and from the Navarre Biobank were explored and reviewed, and MSA diagnosed patients were selected to be included in RERNA. Information on the date of death was accessible and linked to MSA patients in the registry.

The procedures carried out were in accordance with the Helsinki Declaration of 1975, as revised in 2000, and the study was approved by the Navarre Ethical Committee of Clinical Research.

### Ascertainment and data collection

The information from the various data sources was reviewed in order to identify all potential diagnoses of MSA during the study period. Databases were cross-checked to identify duplicate patients, included in more than one data source. Case validation was performed using information from medical records by a neurologist expert in movement disorders. A detailed chart review was carried out, and pertinent information was extracted from each chart. We collected demographic data, date of clinical symptom onset and date of MSA diagnosis. Time to diagnosis was defined as the duration from clinical symptom onset to diagnosis. Detailed clinical features were reviewed that included symptom onset, first diagnosis established by neurologist, and the presence or absence and time of onset of: parkinsonism and levodopa resistant parkinsonism, gait and limb ataxia, cerebellar dysarthria and oculomotor cerebellar features, unexplained voiding difficulties with post-void urinary residual volume ≥ 100 mL, unexplained urinary urge incontinence, orthostatic decrease of blood pressure within 3 min of standing by at least 20 mm Hg systolic or 10 mm Hg diastolic and erectile dysfunction. Additional clinical features were collected that included rapid progression within 3 years of motor onset, moderate to severe postural instability within 3 years of motor onset, cranio-cervical dystonia, severe speech impairment or dysphagia within 3 years of motor onset, pyramidal signs, myoclonic tremor, postural deformities, stridor, cold discoloured hands and feet and pathologic laughter or crying, date of magnetic resonance imaging and radiological findings.

### Diagnostic criteria

Patients were included if they fulfilled the new Movement Disorder Society (MDS) criteria for the diagnosis of clinically established or clinically probable MSA (Online Resource 1). Diagnosis date was defined as the date on which MSA diagnosis was first recorded. We classified MSA subtype depending on the predominant motor variant in MSA-P and MSA-C.

The exclusion criteria of the new MDS MSA diagnostic criteria were applied (Online Resource 1).

### Data analysis

Point prevalence (global and sex-specific) of MSA was calculated annually using the number of MSA individuals per 100,000 inhabitants, resident in Navarre, on December 31. Incidence and mortality rates were defined as the number of newly diagnosed MSA cases and the number of MSA-registered deaths, respectively, per 100,000 inhabitants per year. Mean annual incidence and mortality rates per 100,000 were analysed for two periods: 2012–2016 and 2017–2021. For annual age-adjusted mortality rates, we used the 2013 European Standard Population (ESP) [16] and the 2016 Spanish and Navarre Population [9]. Overall trends were analysed for the three epidemiologic indicators.

Demographic and clinical characteristics were summarized using descriptive statistics, such as median and ranges for continuous variables and absolute and relative frequencies for categorical variables. Continuous variables were compared by sex or MSA subtype using t-test, U Mann–Whitney test for median comparisons and Chi-square test and Fisher’s test for categorical variables. Median survival time and 95% CI were computed using Kaplan–Meier estimators, and comparisons between sexes were performed using the logrank test.

## Results

The search strategy from multiple sources, after removing duplicates and deaths before 2012, produced 819 potential cases that required diagnostic verification (Online resource 2). The clinical diagnosis included Parkinson’s disease and other degenerative Parkinsonism (378), Lewy body dementia and other dementias (155), vascular and drug induced parkinsonism (51), essential tremor (9), hereditary parkinsonism, chorea, and ataxias (107) and 50 miscellanea (functional or unknown origin parkinsonism). Thirteen cases were coding errors. Finally, we identified 56 potential MSA cases. We excluded 16, 5 because enough information was not available from medical records and 11 do not fulfil MSA diagnostic criteria of clinically established or clinically probable MSA. We analysed data from 40 MSA patients (60% male, 57.5% MSA-P subtype).

### Prevalence and incidence

On 31 December 2021, 16 patients were alive giving a crude point prevalence of 2.43 (95% CI 1.24; 3.62) cases per 100,000 inhabitants: 1.2 (95% CI 0.02; 2.38) in women and 3.69 [95% CI (1.60; 5.78)] in men. The mortality rate during the period 2012–2021 was 3.65 person-year/100,000 (95% CI 2.19; 5.11): 3.61 (95% CI 1.57; 5.65) in women and 3.69 (95% CI 1.60; 5.78) in men.

**Fig. 1:**
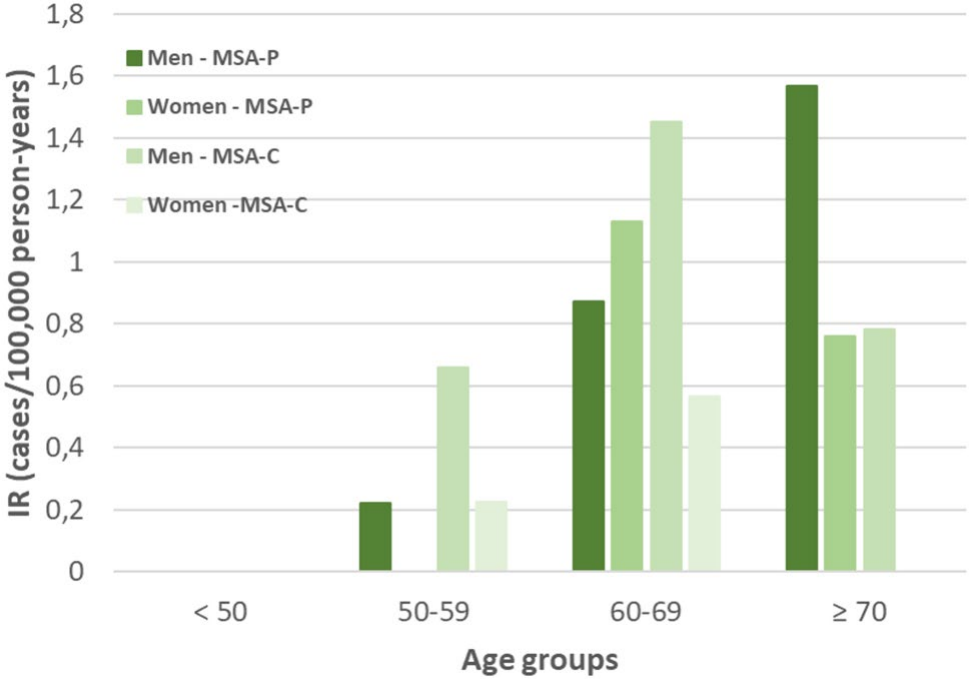
Incidence rates by age groups, multisystem atrophy subtype and sex

Total incident cases were 32 and the crude average annual incidence rate (IR) was 0.49 (0.32; 0.67) per 100,000 person-years in the period 2012–2021 (Table 2). The IR for women was 0.34 (0.14; 0.54) and for men was 0.65 (0.37; 0.93). The incidence adjusted to the World Health Organization standard European population was 0.55 (95% CI 0.36; 0.74). The incidence adjusted to the Spanish population was 0.49 (0.32; 0.65) and adjusted to the Navarre population was 0.49 (0.32; 0.66). Regarding the age-specific annual IR (Fig. 1), the highest incidence occurred in the age group of 60–69 years, with an IR of 2.0 (0.95; 3.06) (Table 2). No significant IR differences by sex or subtype were observed.

**Table 2:** Sex-, age- and period-specific estimates of prevalence and incidence of Multisystem atrophy (MSA) in Navarre, for the period 2012–2021 (diagnosis date)

	Prevalence		Incidence	
	Total prevalent cases	Point prevalence rate per 100,000 (95% CI) persons per year	Total incident cases	Annual incidence rate per 100,000 (95% CI) person-year
Total	40	0.62 (0.43; 0.81)	32	0.49 (0.32; 0.67)
Sex				
Men	24	0.75 (0.45; 1.05)	21	0.65 (0.37; 0.93)
Women	16	0.49 (0.25; 0.73)	11	0.34 (0.14; 0.54)
Age (years at diagnosis)				
< 50	0	–	0	–
50–59	7	0.78 (0.20; 1.35)	5	0.55 (0.07; 1.04)
60–69	17	2.43 (1.28; 3.59)	14	2.0 (0.95; 3.06)
≥ 70	16	1.76 (0.90; 2.62)	13	1.43 (0.65; 2.21)
Period				
2012–2016	21	0.65 (0.37; 0.93)	13	0.40 (0.18; 0.62)
2017–2021	28	0.86 (0.54; 1.18)	19	0.58 (0.32; 0.85)
MSA subtype				
MSA-C	17	0.26 (0.14; 0.39)	14	0.22 (0.10; 0.33)
MSA-P	23	0.36 (0.21; 0.50)	18	0.28 (0.15; 0.41)

*CI* confidence interval, *MSA* multisystem atrophy, *MSA-C* multisystem atrophy cerebellar subtype, *MSA-P* multisystem atrophy parkinsonian subtype

**Fig. 2:**
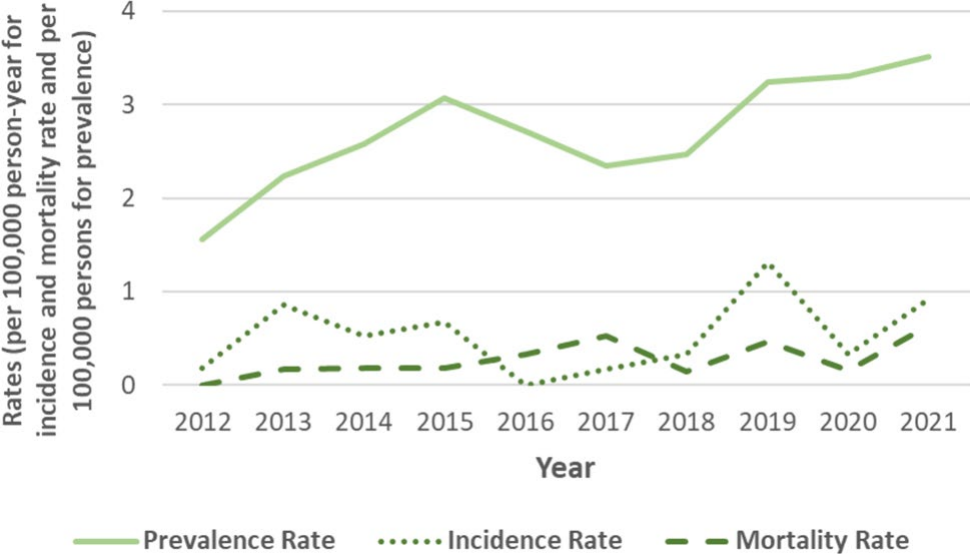
Point prevalence, incidence, and mortality annual rates during the period 2012–2021

Joinpoint analysis for global incidence and prevalence experienced stable annual rates during the whole period, showing an upward trend for prevalence without a statistically significant slop (Fig. 2).

### Clinical features

Main clinical features are summarized in Table 3. The most frequent motor symptoms leading to neurology consultation was gait disturbance or falls (47.5%; 19/40), especially in the MSA-C variant (82.4%; 14/17). REM sleep behaviour disorder was the presenting symptom in 2.5% (1/40) of patients. Unexplained voiding difficulties or urinary incontinence prompted urological consultation before the onset of motor symptoms in 20% (8/40) of patients (75% male and 87.5% MSA-P variant). On evolution, 7.5% (3/40) of patients developed two supporting clinical features (Online Resource 1) and the remaining 92.5% (37/40) developed three or more (range 3–8). Exclusion criteria (Online Resource 1) were absent in all patients. In 10% of patients (4/40), the diagnosis of MSA was established at the first consultation in neurology, all of them corresponding with the MSA-C variant. MRI was normal in 38.09% (8/21) of MSA-P patients and 11.8% (2/17) of MSA-C patients. In 64.28% (18/28) patients various MRI markers were found while isolated MR alterations, in the form of cerebellar or putamen atrophy and putamen increased diffusivity or middle cerebellar peduncle increased diffusivity, were found in 35.7% (10/28) of cases. In 32.5% of patients (13/40), diagnosis was confirmed postmortem, 54.16% (13/24) of those who died. From this group, diagnosis certainty in life was clinically established in 46.15% (6/13) and clinically probable in 53.84% (7/13).

**Table 3:** Clinical features

	Total (n = 40)	MSA-P (n = 23)	MSA-C (n = 17)	P value
Age at onset (years) median (range)	65 (47–76)	68 (47–76)	62 (49–75)	0.015^a^
Sex				
Men n (%)	24 (60)	11 (47.8)	13 (76.5)	ns
Women n (%)	16 (40)	4 (52.2)	12 (23.5)	
Latency-consultation (months) median (range)	6 (1–26)	6 (1–18)	6 (1–26)	ns
Symptoms leading to neurology consultation				
Gait disturbance/falls n (%)	19 (47.5)	5 (21.7)	14 (82.4)	0.0001^b^
Tremor n (%)	5 (12.5)	5 (21.7)	0	
Bradykinesia n (%)	4 (10)	4 (17.4)	0	
Dysarthria n (%)	2 (5)	2 (8.7)	0	
RSBD n (%)	1 (2.5)	0	1 (5.9)	
Combination n (%)	9 (22.5)	7 (30.4)	2 (11.8)	
MRI performed n (%)	38 (95.0)	21 (91.3)	17 (100)	ns
Time MRI (months) median (range)	34 (19–59)	30 (4–126)	35 (2–129)	ns
Level of diagnostic confidence				
Postmortem established n (%)	13 (32.5)	10 (43.5)	3 (17.6)	ns
Clinically established n (%)	17 (42.5)	7 (30.4)	10 (58.8)	
Clinically probable n (%)	10 (25)	6 (26.1)	4 (23.5)	

*Combination* bradykinesia with dysarthria or gait disturbance, *Latency-consultation* time interval from first symptom to first neurology consultation, *MRI* magnetic resonance imaging, *Time MRI* time interval from first symptom to last MRI available study, *ns* non-significant, *RSBD* REM sleep behaviour disorder

^a^U Mann–Whitney test

^b^Chi-Square test (comparing gait disturbance/falls vs. clustering of other symptoms)

### Time to diagnosis and survival

The median duration from onset of symptom to diagnosis was 36 months (95% CI 31.9; 40), without statistically significant differences between sex, age at diagnosis or subtypes (Table 4). The median of survival from clinical symptom onset was 84 months (95% CI 69.03; 98.97) (Table 4) and from clinical diagnosis was 42 months (95% CI 37.0; 47.0), without sex or subtype differences. Kaplan–Meier graphs for time to diagnosis and time from diagnosis onset to death are supplied as supplementary material (Online Resource 3). Age of onset 70 years or older was associated with reduced survival (p < 0.0001). Patients who experienced urinary dysfunction prior to their initial neurology consultation had a significantly lower median survival compared to those who initially presented with motor symptoms (63 months 95% CI 55.30; 70.70) and 86 months (95% CI 64.13; 107.88), respectively (p < 0.01).

**Table 4:** Median of time to diagnosis and time of survival from clinical onset, months (95%CI)

	Median of time to diagnosis, months (95%CI)	p value (log-rank)	Median survival, months (95%CI)	p value (log-rank)
Total	36 (31.9; 40.1)		84 (69.03; 98.97)	
Sex		0.208		0.180
Men	38 (31.3; 44.7)		85 (28.25; 141.75)	
Women	31 (23.2; 38.8)		84 (66.01;101.99)	
Age at diagnosis		0.777		0.004
< 50	–		–	
50–59	27 (23.1;30.8)		134 (118.40; 149.60)	
60–69	34 (25.9; 42.1)		94 (60.36; 127.64)	
≥ 70	37 (35.1; 38.9)		64 (49.97; 78.03)	
MSA subtype		0.972		0.153
MSA-C	36 (21.9; 50.1)		125 (92.05; 157.95)	
MSA-P	37 (33.3; 40.7)		70 (58.63; 81.38)	

*MSA-C* multisystem atrophy cerebellar subtype, *MSA-P* multisystem atrophy parkinsonian subtype

## Discussion

Our study provides a detailed description of age, sex-specific and age-adjusted prevalence, and incidence of MSA in Navarre and represents the first population-based epidemiological study on MSA performed in Spain. An epidemiological study aimed to study Parkinson’s disease prevalence performed in Aragón (Spain) found one case with MSA yielding a crude prevalence of 1.5/100,000 person, thought MSA was not the main objective of the study [17].

Numbers of average annual IR (0.49/100,000 person-years) and age-adjusted prevalence (2.43/100,000) are similar to previous studies with similar methodology conducted in European populations, namely Iceland [18] and Switzerland [5] (Table 1). A recent study carried out in the USA provides much higher incidence and prevalence figures, which we attribute to the methodology employed based on retrospective data retrieval from administrative database without revisions of clinical records [6]. We have not observed differences in incidence over the years, although there is a trend towards an increase in prevalence, which may be due to improved multidisciplinary care and better management of complications. The highest incidence is observed in the 60–69 age group, similarly to the Icelandic study [18] while in Switzerland, the age of onset is lower [5]. Median age at onset is significantly earlier in MSA-C, as observed in other studies [19, 20], and the highest incidence of MSA-P is above 70 years of age, that has also been described in USA [21] and in Scotland [22].

The median of survival in our study was 7 years, similar to previous studies [18, 23, 24]. We have found that patients diagnosed with MSA at the age of 70 and older have significantly shorter survival than those who start at younger ages, in line with other studies [25], and which may be due to co-morbidity at older ages. We found no differences in survival between men and women, neither differences in the time from diagnosis to death, contrary to other studies that described a longer survival from diagnosis in females [20] that was attributed to men consulting later because of more frequent onset of urinary symptoms [23, 26]. However, we found that one-fifth of patients, most of them male, had consulted urology for urinary dysfunction prior to the first neurology consultation, and they had a significantly shorter survival, as has already been reported [26]. It is known that the first symptoms of MSA are frequently autonomic and may predate recognition of motor manifestations [27]. A pre-possible MSA phase has been described, and the improvement in the early diagnosis of MSA seems to depend on the sensitivity and positive predictive values of autonomic features or autonomic function tests [28].

Time to diagnosis in our series is long, median 36 months, with no differences according to subtypes, in line with other European studies [18]. Only in four patients, MSA diagnosis was established at the first neurology visit, reflecting the high degree of clinical overlap that exists in atypical parkinsonism and the difficulty of establishing a precise and early diagnosis in the absence of biomarkers [29].

The limitations of our study are its retrospective nature and the difficulty of diagnostic certainty intrinsic to neurodegenerative diseases. Regarding these points, not only expert neurologists in movement disorder evaluated patients in life and reviewed clinical charts, but also, we strictly applied the new MDS criteria, including the systematic review of MRI. These new MDS criteria have recently been pathologically validated [30] concluding that MRI findings contributed to increase the specificity. We suspect that in our study, most of the patients with probable diagnostic certainty would have reached the clinically established diagnostic level in case of having an MRI in more advanced stages of the disease. These patients met the core clinical features with two or more from any of the 13 supporting features and the absence of exclusion criteria, which has also been shown to provide high diagnostic specificity [31]. In addition, we would like to highlight the high percentage of cases with postmortem diagnostic confirmation in our study. Therefore, the possibility of having included false positives in our study is very low.

We consider that our study has many strengths: recruitment of cases was performed with searching through extensive sources of health information and covers a long period. The Spanish healthcare system involves a high degree of use of public resources. Despite this, the bases of private clinics have also been reviewed. Navarre is a region with a manageable population and with a high density of neurologists, making it very appropriate for epidemiological studies. Therefore, few patients may have been missed.

In conclusion, our work represents the first population-based epidemiological study on MSA in Spain and the first with application of the new MDS diagnostic criteria in Europe. It provides detailed incidence, prevalence, and mortality data for MSA that are in accordance with other European studies and may be useful for the appropriate management of health resources. Comparison with studies from other geographic areas may provide clues to the aetiology of this disease.

## Supplementary Information

Below is the link to the electronic supplementary material.

Supplementary file1 (PDF 126 KB)

Supplementary file2 (PDF 113 KB)

Supplementary file3 (PDF 210 KB)

## Data Availability

The datasets used and analyzed during the current study are available from the corresponding author upon reasonable request.
