# Supplementary material for: Epidemiologic and clinical features of multisystem atrophy: a population-based study in Navarre, Spain
Source: J Neurol. 2024 Aug 13;271(10):6647–54. doi: 10.1007/s00415-024-12561-4 (PMC11446993; doi:10.1007/s00415-024-12561-4)
Supplement: Supplementary file 1 — Supplementary file1 (PDF 126 KB) [file 415_2024_12561_MOESM1_ESM.pdf]

Epidemiologic and clinical features of multisystem atrophy: a population-based study in Navarre, Spain

Erro Aguirre ME[1,2,3,*], Arrondo Gómez P[2], Gastón Zubimendi I[1,3], Clavero Ibarra P[1,3], Sánchez Ruiz de Gordoa J[1,2,3], Martí Andrés G[1], Valentí Azcárate R[4], Delfrade Osinaga J[3,5,6], Vicente E[3,5,6].

[1]Department of Neurology, Hospital Universitario de Navarra, Pamplona, Spain
[2]Epigenetic group, NavarraBiomed. Pamplona, Spain
[3]Navarra Institute for Health Research (IdisNA).
[4]Department of Neurology. Clínica San Miguel, Pamplona, Spain
[5]Community Health Observatory Section, (ISPLN), Pamplona, Spain
[6]CIBER Epidemiology and Public Health (CIBERESP), Madrid, Spain

(*) corresponding author:
María Elena Erro Aguirre, PhD, MD
Neurology Department
Hospital Universitario de Navarra
C/ Irunlarrea 3, 31008 Pamplona (Navarre) SPAIN
Tel.: +34-848-422292
Fax: +34-848-422303
e-mail: elena.erro.aguirre@cfnavarra.es
https://orcid.org/**0000-0002-9707-4190**

Supplementary material. The new Movement Disorders Society criteria for the diagnosis of multisystem atrophy.

**Essential features**	A sporadic, progressive adult (>30 years) onset disease	
	**Clinically established**	**Clinically probable**
**Core clinical features**	Autonomic dysfunction defined as (**at least one** is required)<br>• Unexplained voiding difficulties with post-void urinary residual volume ≥100 mL<br>• Unexplained urinary urge incontinence<br>• Neurogenic OH (≥20/10 mmHg blood pressure drop) within 3 minutes of standing or head-up tilt test	**At least two of:**<br>1. Autonomic dysfunction defined as (at least one is required):<br>• Unexplained voiding difficulties with post-void urinary residual volume<br>• Unexplained urinary urge incontinence<br>• Neurogenic OH (≥20/10 mmHg blood pressure drop) within 10 minutes of standing or head-up tilt test<br>2. Parkinsonism<br>3. Cerebellar syndrome (at least one of gait ataxia, limb ataxia, cerebellar dysarthria, or oculomotor features)
	+	
	**At least one** of<br>• Poorly L-dopa-responsive parkinsonism<br>• Cerebellar syndrome (at least two of gait ataxia, limb ataxia, cerebellar dysarthria, or oculomotor features)	
**Supportive features (motor or non motor)**	**Al least two**	**At least one**[a]
**MRI marker**	**Al least one**	Non required
**Exclusion criteria**	Absence	Absence
	**Supportive motor features**	**Supportive Non-motor features**
	Rapid progression within 3 years of motor onset<br>Moderate to severe postural instability within 3 years of motor onset<br>Craniocervical dystonia induced or exacerbated by L-dopa in the absence of limb dyskinesia<br>Sever speech impairment within 3 years of motor onset<br>Severe dysphagia within 3 years of motor onset<br>Unexplained Babinski sign<br>Postural deformities<br>Jerky myoclonic postural or kinetic tremor	Stridor<br>Inspiratory sighs<br>Cold discolored hands and feet<br>Erectile dysfunction (below age of 60 years for clinically probable MSA)<br>Pathologic laughter or crying
**MRI markers of clinically established MSA**	For MSA-P	For MSA-C
	• Atrophy of:	• Atrophy of:

	Putamen (and signal decrease on iron-sensitive sequences)<br>Middle cerebellar peduncle<br>Pons<br>Cerebellum<br>• “Hot cross bun” sign	Putamen (and signal decrease on iron-sensitive sequences)<br>Infratentorial structures (pons and middle cerebellar peduncle)<br>• “Hot cross bun" sign<br>• Increased diffusivity of: Putamen
	**Exclusion criteria**	
	Substantial and persistent beneficial response to dopaminergic medications<br>Unexplained anosmia on olfactory testing<br>Fluctuating cognition with pronounced variation in attention and alertness and early decline in visuoperceptual abilities<br>Recurrent visual hallucinations not induced by drugs within 3 years of disease onset<br>Dementia according to DSM-V within 3 years of disease onset<br>Downgaze supranuclear palsy or slowing of vertical saccades<br>Brain MRI findings suggestive of an alternative diagnosis (eg, PSP, multiple sclerosis, vascular parkinsonism, symptomatic cerebellar disease, etc.)<br>Documentation of an alternative condition (MSA look-alike, including genetic or symptomatic ataxia and parkinsonism) known to produce autonomic failure, ataxia, or parkinsonism and plausibly connected to the patient’s symptoms a Excluding erectile dysfunction as an isolated feature.	

a Excluding erectile dysfunction as an isolated feature.

Abbreviations: MSA, multiple system atrophy; MSA-P, MSA-parkinsonian type; MSA-C, MSA-cerebellar type; OH, orthostatic hypotension; MRI, magnetic resonance imaging; DSM-V, Diagnostic and Statistical Manual of Mental Disorders, Fifth Edition; PSP, progressive supranuclear palsy.

Adapted from Weninng GK et al.[4]
