# Supplementary material for: Epidemiologic and clinical features of multisystem atrophy: a population-based study in Navarre, Spain
Source: J Neurol. 2024 Aug 13;271(10):6647–54. doi: 10.1007/s00415-024-12561-4 (PMC11446993; doi:10.1007/s00415-024-12561-4)

Epidemiologic and clinical features of multisystem atrophy: a population-based study in Navarre, Spain

Erro Aguirre ME[1,2,3,*], Arrondo Gómez P[2], Gastón Zubimendi I[1,3], Clavero Ibarra P[1,3], Sánchez Ruiz de Gordoa J[1,2,3], Martí Andrés G[1], Valentí Azcárate R[4], Delfrade Osinaga J[3,5,6], Vicente E[3,5,6].

[1]Department of Neurology, Hospital Universitario de Navarra, Pamplona, Spain
[2]Epigenetic group, NavarraBiomed. Pamplona, Spain
[3]Navarra Institute for Health Research (IdisNA).
[4]Department of Neurology. Clínica San Miguel, Pamplona, Spain
[5]Community Health Observatory Section, (ISPLN), Pamplona, Spain
[6]CIBER Epidemiology and Public Health (CIBERESP), Madrid, Spain

(*) corresponding author:
María Elena Erro Aguirre, PhD, MD
Neurology Department
Hospital Universitario de Navarra
C/ Irunlarrea 3, 31008 Pamplona (Navarre) SPAIN
Tel.: +34-848-422292
Fax: +34-848-422303
e-mail: elena.erro.aguirre@cfnavarra.es
https://orcid.org/**0000-0002-9707-4190**

Supplementary material. Kaplan-Meier graphs for time to diagnosis and time from diagnosis onset to death of multisystem atrophy (MSA).

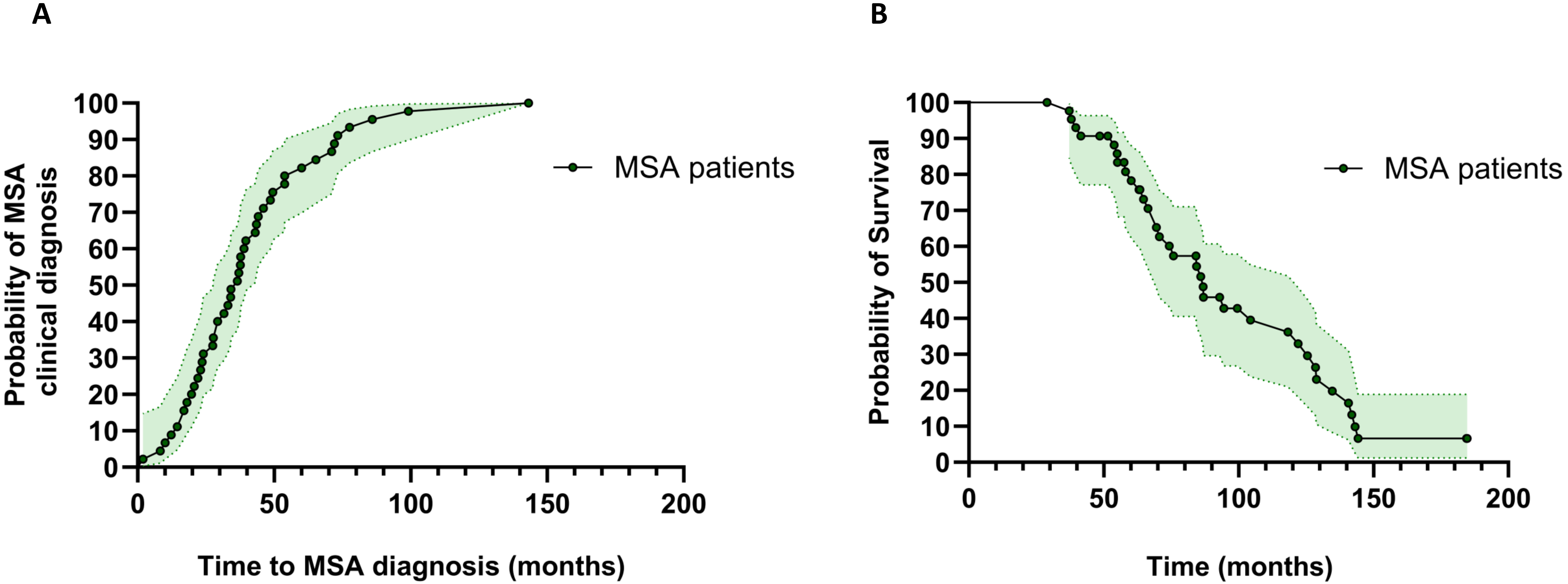

Supplement: Supplementary file 3 — Supplementary file3 (PDF 210 KB) [file 415_2024_12561_MOESM3_ESM.pdf]
